# Theta Oscillation Reveals the Temporal Involvement of Different Attentional Networks in Contingent Reorienting

**DOI:** 10.3389/fnhum.2016.00264

**Published:** 2016-06-03

**Authors:** Chi-Fu Chang, Wei-Kuang Liang, Chiou-Lian Lai, Daisy L. Hung, Chi-Hung Juan

**Affiliations:** ^1^Institute of Cognitive Neuroscience, National Central UniversityTaoyuan City, Taiwan; ^2^Department of Neurology, Kaohsiung Medical UniversityKaohsiung City, Taiwan

**Keywords:** contingent reorienting, attentional networks, theta oscillation, inter-trial coherence, N2pc

## Abstract

In the visual world, rapidly reorienting to relevant objects outside the focus of attention is vital for survival. This ability from the interaction between goal-directed and stimulus-driven attentional control is termed contingent reorienting. Neuroimaging studies have demonstrated activations of the ventral and dorsal attentional networks (DANs) which exhibit right hemisphere dominance, but the temporal dynamics of the attentional networks still remain unclear. The present study used event-related potential (ERP) to index the locus of spatial attention and Hilbert-Huang transform (HHT) to acquire the time-frequency information during contingent reorienting. The ERP results showed contingent reorienting induced significant N2pc on both hemispheres. In contrast, our time-frequency analysis found further that, unlike the N2pc, theta oscillation during contingent reorienting differed between hemispheres and experimental sessions. The inter-trial coherence (ITC) of the theta oscillation demonstrated that the two sides of the attentional networks became phase-locked to contingent reorienting at different stages. The left attentional networks were associated with contingent reorienting in the first experimental session whereas the bilateral attentional networks play a more important role in this process in the subsequent session. This phase-locked information suggests a dynamic temporal evolution of the involvement of different attentional networks in contingent reorienting and a potential role of the left ventral network in the first session.

## Introduction

Things that are of importance or immediate relevance easily capture our attention even when they appear out of the corner of our eyes. For instance, when we search for a friend wearing a red coat in a crowd, we can efficiently filter out people without this feature but may occasionally get distracted by someone with a red hat. This phenomenon is an example of contingent capture, where the focus of attention is shifted to other objects that match the current goal of the observer (Folk et al., 1992, 2002; Theeuwes, 2010). Such a process is characterized as the results of involuntary attentional reorienting and the integration between top-down and stimulus-driven attentional control. This characteristic of rapidly reorienting to any object that shares a target-defining feature is, although sometimes error-prone, an essential and advantageous aspect of attention which helps to increase the efficiency of visual search.

Neuroimaging studies have demonstrated that two discrete attentional networks, which are distributed in the fronto-parietal regions, are simultaneously activated during contingent reorienting (Corbetta and Shulman, 2002; Corbetta et al., 2008). The dorsal attentional network (DAN) is mainly responsible for goal-driven or top-down attentional control (Nobre, 2001; Yantis et al., 2002; Rushworth and Taylor, 2006). The ventral attentional network (VAN), with the co-activation of the DAN, is specific for contingent capture (Serences et al., 2005; Diquattro et al., 2013). However, neuroimaging studies measuring hemodynamic responses lack sufficient temporal resolution to investigate the temporal dynamics of these attentional networks. It has been shown from previous behavioral studies that contingent capture varies across location and time. The effect of contingent capture shows asymmetry in the left and right visual fields (RVFs; Du and Abrams, 2010; Burnham et al., 2011) and it can also be suppressed after repeated exposure to the distractors (Zehetleitner et al., 2012). Such variations in behavioral performance reflect the adaptive characteristics of the underlying neural mechanisms, especially the dynamic interaction between the VAN and DAN. In order to elucidate the involvement of the VAN and DAN during contingent capture, measurement techniques with high temporal resolution is highly essential.

The aim of the present study was to use electroencephalography (EEG) and phase-locking information to investigate the temporal evolution of contingent reorienting and the involvement of the underlying brain regions in this process. In previous visual attention research using event-related potential (ERP), the N2pc component (the negative waveform recorded on the posterior scalp that is contralateral to the attended visual field) has been proved to be a reliable electrophysiological marker of covert spatial attention (Luck and Hillyard, 1994; Woodman and Luck, 2003). Some studies showed that contingent capture elicits similar electrophysiological activity associated with target processing (Sawaki and Luck, 2011; Kiss et al., 2012), which can be indexed by N2pc. Given that N2pc is a lateralized component, we used the rapid serial visual presentation (RSVP) paradigm and systematically manipulated the location of distractors to better control the attentional processing involved in contingent reorienting (Serences et al., 2005; Chang et al., 2013). The RSVP was designed to separate the target and distractors in space and time. Because the target was at the center of the display and the distractors appeared only to the left and right of the target and before target onset, these spatial and temporal configurations allowed us to investigate contingent reorienting caused by the flanking distractors. In this case, the lateralized ERP components would be mainly invoked by the lateral distractors but not the central target. Thus, the magnitude of contingent capture could be quantified by the amplitude of N2pc, which can be used to examine the potential temporal evolution and visual asymmetry of contingent reorienting.

In addition to analyzing ERP components, another way to utilize electrophysiological signals is to extract neural oscillatory information to better understand the interaction between brain regions involved in contingent reorienting. Neural oscillatory activity reflects the intrinsic property of neuronal activity (e.g., Buzsáki and Draguhn, 2004). One functional role of neuronal oscillation is thought to be related to the communication between neural populations (Engel et al., 2001; Fries, 2005). Low-frequency oscillation is mainly in charge of long-range communication between brain regions, whereas high-frequency oscillation in charge of local field potentials (Stein et al., 2000). Some electrophysiological studies showed that theta oscillation is highly related to the N2pc component when spatial attention shifts to a peripheral distractor (Dowdall et al., 2012; Painter et al., 2014). Moreover, a recent electrocorticography (ECoG) recording study demonstrated a transient phase resetting in the theta oscillation following attentional reorienting to the unexpected target (Daitch et al., 2013). This evidence clearly demonstrates that theta oscillation is crucial for the orienting of spatial attention.

However, past studies that investigated neural oscillation mostly used the linear and stationary additive Fourier analysis, which has great limitations for analyzing brainwaves as they are characterized by nonlinear and nonstationary signals (Stam, 2005). An appropriate method to extract time-frequency information from the original EEG signals is the Hilbert-Huang transform (HHT) developed by Huang et al. (1998). Unlike the Fourier and Wavelet analysis, the HHT was designed to adaptively decompose nonstationary and nonlinear signals into a set of intrinsic oscillatory modes, which provides better signal-to-noise ratio for EEG or ERP analysis (Williams et al., 2011) and has already been demonstrated to be a promising method for the analysis of neurobiological time-series data (Liang et al., 2005). In visual attention studies, Desimone and colleagues have also applied HHT to acquire the instantaneous local field potential power and demonstrated that attention modulates the local field potential power (Gregoriou et al., 2009, 2012).

Another advantage of the HHT is that it provides better temporal and frequency resolution than Fourier-based analysis (Huang and Wu, 2008). With instantaneous temporal resolution in amplitude, frequency and phase, the HHT is particularly suited to the goal of the current study to investigate the transient modulation in neural activity during attentional reorienting. In the current study, we applied HHT to our time-frequency analysis to elucidate the temporal and spatial modulation in attentional reorienting. We also reconstructed the source of the instantaneous scalp distribution in theta band to investigate the critical oscillatory activity in attentional reorienting to reveal the communication between the underlying brain regions. Given that previous studies have shown that attentional reorienting is mainly related to theta oscillation (Dowdall et al., 2012; Daitch et al., 2013; Painter et al., 2014), we predicted that contingent reorienting would also be associated with theta activity. We hope that, by revealing the temporal evolution of contingent reorienting and its adaptive and dynamic characteristics, the present study will shed new light on the interaction between attentional networks at the neural oscillatory level.

## Materials and Methods

### Participants

Twenty-nine neurologically healthy university students with normal or corrected-to-normal vision participated in the experiment (17 male, 12 female; mean age = 21.97 years, SD = 1.68 years). All gave informed consent prior to participation. All experimental procedures were approved by the Institutional Review Board of the Kaohsiung Medical University Chung-Ho Memorial Hospital, Kaohsiung, Taiwan.

### Stimuli and Task

**Figure 1:**
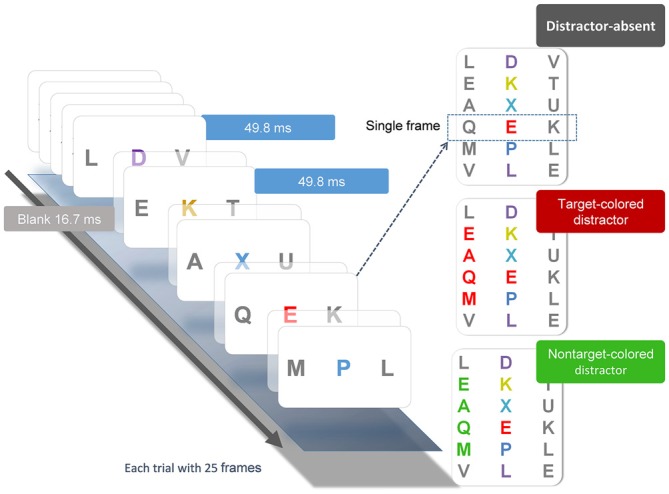
**Illustration of the experimental conditions and procedure.** Each stream consisted of 25 frames of three-letter sequences presented in serial order. Each frame was presented for 49.8 ms, followed by a 16.7 ms blank interval. Participant searched for the unique red letter in the central stream. The peripheral flanker streams were mostly gray letters. On one-third of the trials, the target-colored (TC) distractors were presented in the flanker streams two frames before the onset of the central target and lasted for four frames after the offset of the target. On another one third of the trials, these distractors were in a nontarget color. On the remaining one third of the trials, the distractors were all gray.

The behavioral task (Figure 1) was a modification from Serences et al. (2005) and was used in one of our previous studies (Chang et al., 2013). Participants pressed the space bar to initiate each trial, which began with a 500-ms black fixation cross at the center of the screen, followed by a RSVP of three-letter sequences. Each stream consisted of 25 frames. Each frame contained three uppercase letters and was presented for 50 ms, followed by a 16.7 ms blank interval, yielding a rate of 66.7 ms/frame. In all letter streams, each letter subtended 1° horizontally and 1.3° vertically. The letters were selected randomly without replacement from the English alphabet. Participants were required to identify the red (Commission International del; Eclairage, *x* = 0.62, *y* = 0.32) letter (i.e., the target) embedded in the central stream of other colored letters (i.e., the distractors), which were randomly chosen between yellow (*x* = 0.38, *y* = 0.46), blue (*x* = 0.17, *y* = 0.08), and purple (*x* = 0.25, *y* = 0.11). Colors in the array were approximately isoluminant (26 cd/m^2^). Across trials, the target appeared randomly between the 11th and 14th frames and was selected equally from the first eight and last eight of the English alphabet (A to H, S to Z). After each stream, participants responded by pressing a button with their right index finger if the target belonged to the first eight letters of the alphabet, or another button with their right middle finger if the target belonged to the last eight letters. Accuracy was emphasized and speeded responses were not necessary.

Flanking the target stream were two distractor letter streams located 3° to the left and right of the central stream. On one-third of the trials, the colors of both peripheral distractor streams were gray (*x* = 0.32, *y* = 0.34). On the other two-thirds of the trials, in either the right or left letter stream, four of the peripheral distractor letters would change color to either red or green (equally likely). The red distractors shared the same color as the target and are categorized as target-colored (TC) distractors, whereas the green distractors are categorized as nontarget-colored (NTC) distractors. The colored distractors were presented 100 ms before the onset of the central target and lasted for 50 ms after the offset of the target (four frames). Participants were instructed to continuously maintain fixation on the central target stream and ignore the peripheral distractor streams. The duration of the behavioral task lasted between 20–25 min, which varied with subjects’ response times. Each subject completed two sessions on the same day. Each session contained 120 trials.

### EEG Protocol

EEG activity was recorded with Ag/AgCl electrodes mounted in an elastic cap (Electrocap International) using a 36-electrode arrangement following the International 10–20 System (FP1, FP2, F7, F3, Fz, F4, F8, FT7, FC3, FCz, FC4, FT8, T3, C3, Cz, C4, T4, TP7, CP3, CPZ, CP4, TP8, T5, P3, Pz, P4, T6, O1, Oz, O2, HEOL, HEOR, VEOU, VEOL, A1, A2), offline referenced to the left and right mastoid. Vertical and horizontal electro-oculograms (EOG) were also recorded. Impedances of all electrodes were kept below 5 kΩ. Data were recorded with Neuroscan 4.2 Software, with a sampling rate of 1000 Hz and bandpass filters between 0.05 and 70 Hz.

### ERP Analysis

The N2pc is the primary ERP marker for the analysis, which represents the allocation of spatial attention. All ERP analyses after data acquisition were conducted using EEGLAB Toolbox (Delorme and Makeig, 2004) and ERPLAB Toolbox^1^. The onset of the distractor was set as the zero point and the continuous EEG data were epoched from −100 to 600 ms. Artifact rejection was performed to exclude trials with eye blinks (EOG amplitude > ±50 μV) or with movement artifacts (EEG > ±100 μV). Three subjects were excluded because more than 40% of the total trials were rejected (average rejection rate was 12.33% and the standard deviation of rejection rate was 12.6%) and one subject’s EEG data were missing (due to unintended machine malfunction). Data from 25 subjects were included and analyzed. The N2pc was computed as the deflection of the ERP waveform, at posterior sites contralateral to the distractor location (Sawaki et al., 2012). P3 and P4 electrodes were selected as the posterior sites and amplitude difference was computed by subtracting the ipsilateral site from the contralateral site. The time window of N2pc was between 160 and 240 ms.

### Hilbert-Huang Transform (HHT)

To extract time-frequency information from the EEG signals, the HHT was applied to the continuous EEG data from −300 to 755 ms relative to the onset of distractor. The HHT consists of empirical mode decomposition (EMD) and Hilbert spectral analysis transform (Huang et al., 1996, 1998). First, EMD sequentially decomposes a signal into the sum of a finite number of intrinsic mode functions (IMFs). Each IMF is decomposed with the following definitions: (1) the number of local extrema (including local maxima and local minima) and the number of zero-crossings must either be equal or differ at most by 1; and (2) the mean value of the envelope defined by the local maxima and the envelope defined by the local minima is 0. The IMFs represent different oscillatory modes contained in the data. Here an ensemble EMD (EEMD), a noise-assisted version of EMD was applied for each trial to resolve the mode-mixing problem that the original EMD method might cause. In addition, EEMD has also been shown to be more resistant to noises than EMD (Huang and Wu, 2008; Chen et al., 2010) and it provided a remarkably high signal-to-noise ratio and strong effect size when a small number of trials were used in an ERP study of mismatch negativity (Hsu et al., 2016).

To apply EEMD, the IMFs are generated from ensemble means of trials by repeating EMD on the same signal with different sets of Gaussian noise. The current EEMD analysis was applied with 10 times of sifting and 40 ensembles. For each time of each ensemble, the amplitude of the Gaussian noise was 10 percentage of the EEG segment’s standard deviation. The current analysis will focus on the IMF located in the theta band. Second, the Hilbert spectrum was calculated for each trial and each IMF to acquire the instantaneous information about frequency, amplitude and phase. The HHT was applied with customized MATLAB (MathWorks) scripts with ensemble EMD code provided by the Research Center for Adaptive Data Analysis of National Central University, Taiwan^2^. Further data processing and statistical analysis was performed using SPM8 for MEG/EEG (Wellcome Department of Cognitive Neurology, London, UK^3^).

To investigate whether contingent reorienting induces phase modulation, inter-trial coherence (ITC) was computed from theta IMF in the sensor and source level. ITC reflects the consistency of oscillatory phase across trials at a particular latency and it is equivalent to the magnitude of the mean resultant vector of the oscillatory phase across trials. If phase across trials uniformly distributes between 0 and 2π, the ITC will be 0. In contrast, the ITC will be equal to 1 if a phase distribution completely concentrates at the same direction. Larger ITC indicates that trials are phase-locked to each other. ITC was computed at each time point within a trial by the instantaneous phase from HHT results.

Statistical tests were performed on mean IMFs and ITC using a cluster-based non-parametric permutation (CBnPP) test (Maris and Oostenveld, 2007; Groppe et al., 2011) to test the differences of multi-channel amplitude between two distractor-present conditions (TC vs. NTC distractors). The purpose of using the contrast between TC and NTC was to minimize the effect of peripheral sensory change and the NTC condition was used as a baseline in our analysis. Originally, this permutation method was used to provide weak family-wise error rate (FWER) control for EEG- and MEG-data by grouping test results at nearby sensors and time points into clusters based on their statistical significance and proximity. In the current experiment, two EEG sensors/sources were identified as neighbors if the distance between both was less than 40 mm/12.5 mm, and 2000 permutations were performed for each test. This method not only has the advantage of protecting against multiple comparison errors, but it is also powerful (less conservative, in comparison with the Bonferroni or false discovery rate correction) to reveal significant effects, especially for the clustered effect like that from EEG data.

### Source Reconstruction

To investigate the sources activity of the scalp theta IMF, we applied a beamforming approach based on an adaptive spatial filtering technique (Gross et al., 2001; Schoffelen et al., 2008). All linear beamforming analyses were performed using the FieldTrip toolbox^4^ and custom MATLAB scripts. For the beamforming analysis, the conventional practice applied narrow band bandpass filters to the signal before beamforming. The results of EEMD can be considered as a dyadic filter bank structure which is similar to the results of bandpass filters (Flandrin et al., 2004), thus the theta IMF with narrow frequency range satisfies the requirement of beamforming analysis, as the conventional filtered frequency band. The theta IMF from previous HHT analysis in sensor level was used with 1056 ms data segment (−300 to 755 ms, 0 as distractor onset).

A realistically shaped 3-shell head model was derived from the Montreal Neurological Institute (MNI) template brain (Colin 27). The brain volume of the template brain was divided into a grid with a 12.5 mm resolution, resulting in 1540 grid points (898 grid points were within the volume of cortex). The lead fields for each grid point were calculated using the boundary element method (BEM). To obtain the source-level theta activity, a virtual electrode approach was used—applying linearly constrained minimum variance (LCMV) adaptive spatial filtering beamformer (Van Veen et al., 1997) to the sensor-level. The spatial distribution of power was estimated for each trial separately and then averaged per condition and contrasted in order to reduce the center bias. The dipole orientation at each location was determined by finding the projection of the lead field that beamforms the maximum power (Sekihara et al., 2004). After the theta IMF was reconstructed in the source level, the Hilbert transform was applied to the source-level theta IMF to acquire the instantaneous phase information. The source-level ITC was computed with the same method as sensor level. The CBnPP was performed on the ITC results from all grid points to estimate the ITC change for the difference between TC and NTC conditions across subjects.

## Results

### Behavioral Performance

**Figure 2:**
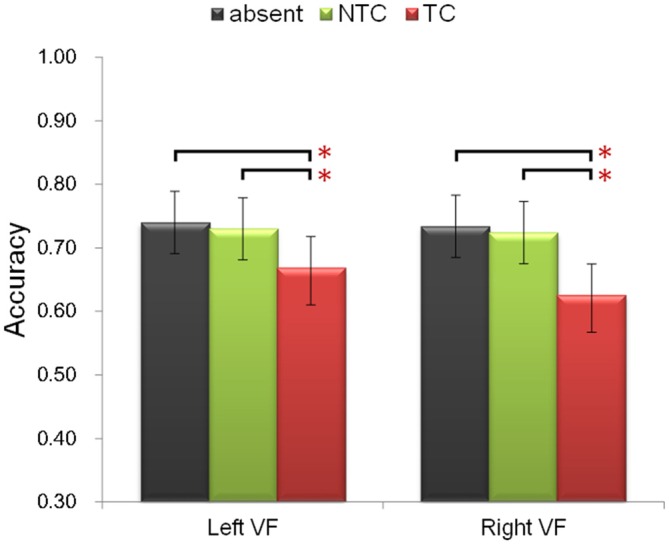
**Illustration of the behavioral performance.** The contingent capture effect (lower accuracy of the TC distractor condition, denoted by the red bars) was equal in both visual fields and different experimental sessions. Error bars represent 95% confidence intervals. *p < 0.05.

Participants were asked to identify the sole red letter in the RSVP task. The following ANOVA was performed for accuracy. Firstly, to include all three factors of the experimental design, a three-way repeated-measures ANOVA was performed with factors of experimental session (first, second), distractor type (absent, NTC, TC), and distractor location (left, right). There was a significant main effect of distractor type [*F*_(2,48)_ = 20.74, *p* < 0.001, *η*^2^ = 0.464]. *Post hoc* paired-sample *t*-test comparisons confirmed previous behavioral results (Folk et al., 1992; Lamy et al., 2004; Serences et al., 2005): accuracy in the TC distractor condition was lower than that in the NTC condition [*t*_(24)_ = 4.571, *p* < 0.001] and colored-distractor absent condition [*t*_(24)_ = 5.358, *p* < 0.001] (Figure 2). There was no difference between the NTC condition and colored-distractor absent condition [*t*_(11)_ = 0.968, *p* = 0.376]. In addition, participants’ accuracy in the second session was higher than that in the first session [*F*_(1,24)_ = 10.857, *p* < 0.01, *η*^2^ = 0.311, 68.8% vs. 72.0%]. The main effect of distractor location and all the other interactions were not statistically significant (*p*s > 0.05).

### ERP Analysis

**Figure 3:**
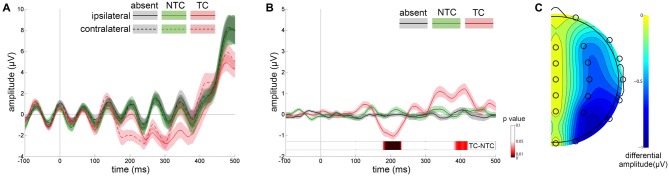
**Results of event-related potential (ERP) waveforms and N2pc scalp distribution map. (A)** Grand average waveforms for each distractor condition at contralateral vs. ipsilateral P3/P4 electrode sites. **(B)** The difference between the contralateral and ipsilateral waveforms. The differential waveforms showed that only TC distractors (red line) induced significant N2pc in comparison with the distractor-absent (gray line) and nontarget-colored (NTC) distractor (green line) conditions. Error shading indicates SEMs. Permutation test results for the contrast of the TC vs. NTC are presented at the bottom. **(C)** Scalp topography during the N2pc time window (160–240 ms) created by the differential amplitude between contralateral and ipsilateral waveforms.

Figure 3A shows the ERP waveforms from the lateral occipital scalp sites (P3 and P4) for distractor types averaged across participants. Separate waveforms are shown for contralateral and ipsilateral sites, relative to the location of distractors. Figure 3B shows the difference between contralateral and ipsilateral waveforms. A three-way repeated-measures ANOVA was computed based on the mean amplitudes of the N2pc with factors of experimental session (first, second), distractor type (absent, NTC, TC) and recording site (P3, P4). The main effect of distractor type was significant [*F*_(2,48)_ = 23.285, *p* < 0.001, *η*^2^ = 0.492] and the N2pc amplitude in the TC distractor condition was larger than that in the other two conditions [vs. absent condition: *t*_(24)_ = 4.478, *p* < 0.001, vs. NTC condition: *t*_(24)_ = 6.118, *p* < 0.001]. We did not observe significant interactions between distractor type and the other two factors (*p*s > 0.05), nor was there a significant main effect of recording site [*F*_(1,24)_ = 1.496, *p* > 0.05, *η*^2^ = 0.059] or experimental session [*F*_(1,24)_ = 1.209, *p* > 0.05, *η*^2^ = 0.048]. The topographic map of the N2pc component is plotted in Figure 3C.

Apart from the N2pc time window, the contralateral-minus-ipsilateral waveform also showed significant differences before and after N2pc. The mean amplitude of these two time windows (before N2pc: 80–160 ms, after N2pc: 240–500 ms) were computed and submitted to two separate ANOVAs with the same factors as above. The results showed that the main effect of distractor type was significant [before N2pc: *F*_(2,48)_ = 6.288, *p* < 0.01, *η*^2^ = 0.208; after N2pc: *F*_(2,48)_ = 6.655, *p* < 0.01, *η*^2^ = 0.217]. The *post hoc* tests showed that the amplitude of the TC condition in both time windows were significantly different from that in the distractor-absent condition [before N2pc: *t*_(24)_ = 3.662, *p* < 0.01; after N2pc: *t*_(24)_ = −2.662, *p* < 0.05]. An additional permutation *t*-test for the contrast of the TC vs. NTC conditions revealed a significant amplitude difference occurred during the time windows of 178–231 ms and 380–421 ms after distractor onset (Figure 3B).

### HHT Analysis

**Figure 4:**
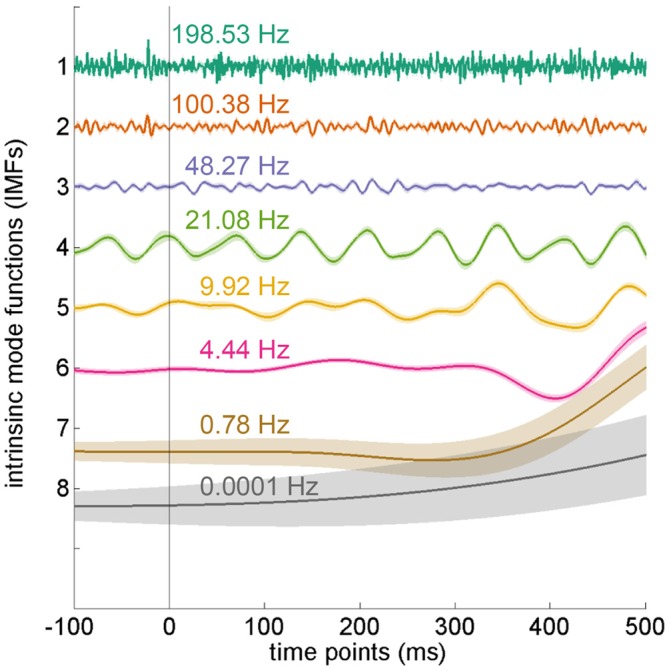
**Intrinsic mode functions from ensemble empirical mode decomposition (EEMD).** The electroencephalography (EEG) data were decomposed into eight intrinsic mode functions (IMFs) by using EEMD. Each IMF denotes a different frequency band. The IMFs depicted in the figure were averaged from all electrodes in the distractor-absent condition and arranged by frequency. The theta oscillation is located at the sixth IMF. Error shading indicates SEMs.

After applying EEMD, the EEG data were transformed into eight IMFs, from high to low frequencies (Figure 4). In the current experiment, we mainly focused on the theta frequency, which was located at the sixth IMF with an average frequency of 4.44 Hz (SD = 0.15 Hz).

**Figure 5:**
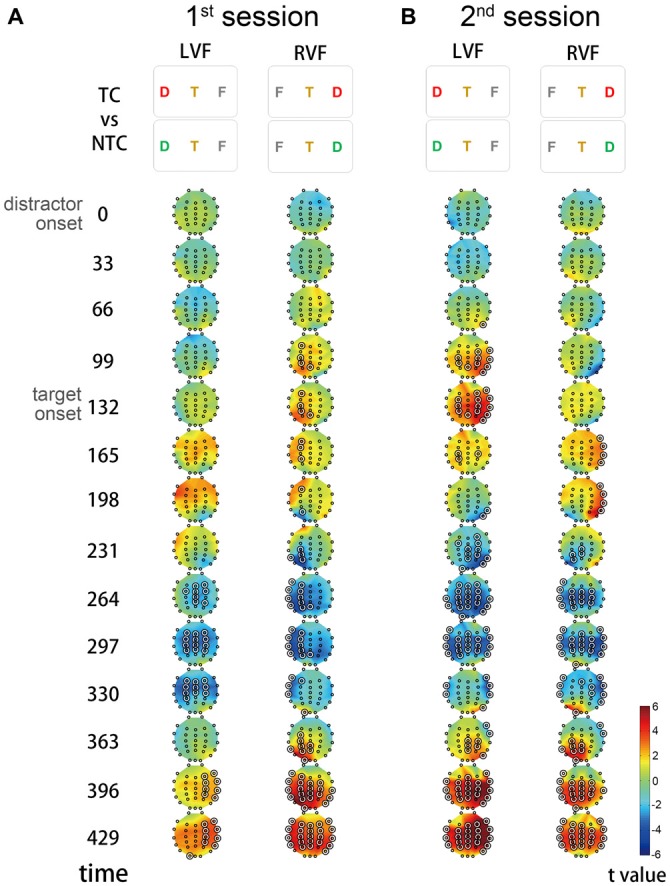
**Scalp distribution of the theta IMF amplitude.** The *t* value denotes the comparison between the TC and NTC distractor conditions. Each topographic frame denotes the instantaneous time point and is illustrated from 0 to 429 ms relative to the onset of distractor. The inter-stimulus interval of rapid serial visual presentation (RSVP) was 66.4 ms, so every two topographic frames equal an RSVP frame. The permutation results showed that significant theta modulation started from the left hemisphere and then propagated to the right hemisphere. In the first session, the theta amplitude increased from the left hemisphere when the TC distractors appeared in the right visual field (RVF) **(A)**. In contrast, the theta amplitude increased from the right hemisphere in the second session **(B)**. The highlighted concentric circles in the topographic maps denote *p* < 0.05 in the cluster-based non-parametric permutation (CBnPP).

To better understand the scalp distribution and the dynamic modulation of the theta IMF, we applied a CBnPP test on the amplitude of the theta IMF (Maris and Oostenveld, 2007) and used the contrast of TC vs. NTC distractor conditions in different sessions and visual fields to isolate contingent reorienting from the processing of peripheral sensory events. Generally, the CBnPP test showed that the amplitude of the theta IMF was larger in the TC distractor condition than in the NTC distractor condition and the increased amplitude was specific for contralateral stimuli (Figure 5). Similar to the ERP results, the amplitude of the theta IMF was more negative during the time window of N2pc and a positive-going wave was observed both before and after the negative wave. These waves all originated from the posterior scalp.

A noteworthy difference was that the first positive peak varied with session and visual field. The positive amplitude was only significantly larger in the left hemisphere (i.e., when the distractors were in the RVF) in the first session but shifted to the right hemisphere (i.e., when the distractors were in the left visual field (LVF)) in the second session.

The subsequent negative-going and positive-going differences observed were consistent with our contralateral-minus-ipsilateral waveform analysis above and did not differ between sessions and visual fields regardless of the location of the distractors.

In addition, we performed cross-subjects correlations to investigate the relationship between N2pc and theta amplitude. In the correlation analysis, the peak amplitude of N2pc was computed by the time window which was decided by the permutation results (178–231 ms). In order to compare with the results of N2pc, the same contralateral electrodes were used to compute the amplitude of theta IMF in the second negative waveform, which shared a similar pattern in the direction of waveform and overlapping time windows. The time windows of the negative theta amplitude were also decided by the permutation results (1st session: 193–312 ms; 2nd session: 215–276 ms).

In the first step, the correlations between the N2pc amplitude and negative theta amplitude were computed for different sessions, visual fields and distractor conditions. The results did not reveal any significant correlation (*p* > 0.1). In the second step, we used the differential amplitude between the TC and NTC distractor conditions for the correlation analysis. The results did not reveal any significant correlation either (*p* > 0.1).

### Inter-Trial Coherence

**Figure 6:**
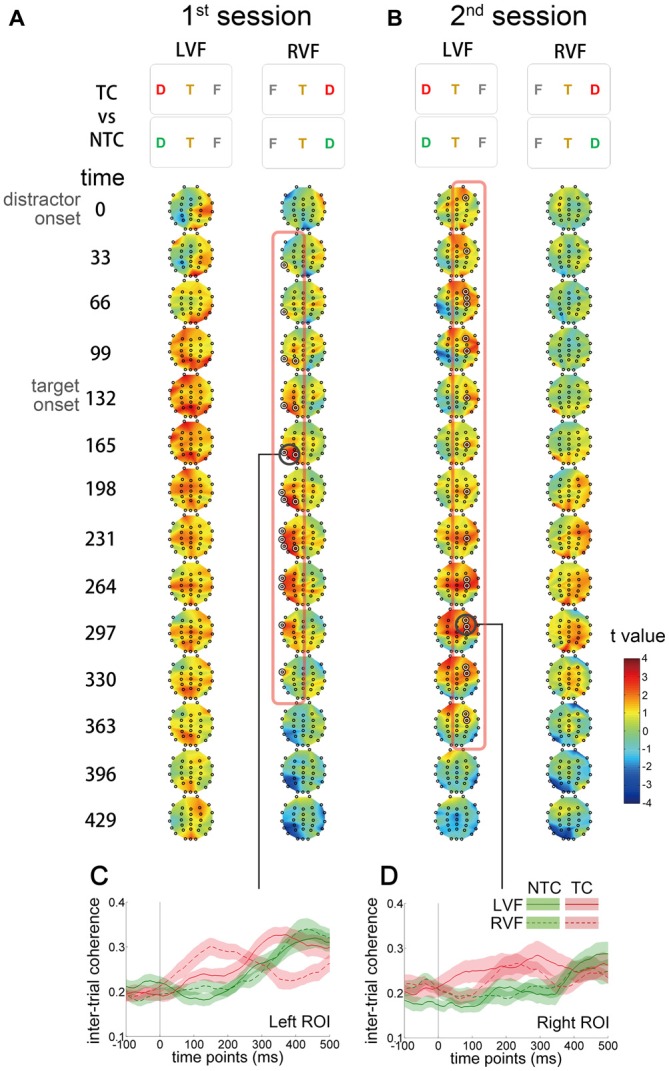
**Scalp distribution and temporal profiles of theta IMF inter-trial coherence (ITC).** The *t* value denotes the comparison between the TC and NTC distractor conditions. Each topographic frame denotes the instantaneous time point and is illustrated from 0 to 429 ms relative to the onset of distractor (separated every 33 ms). The inter-stimulus interval in the RSVP stream was 66.4 ms, so every two topographic frames equal an RSVP frame. **(A)** In the first session, ITC increased in the left temporal and parietal regions after the onset of RVF distractors to 300 ms after onset. **(B)** In the second session, ITC increased in the right frontal region in the left visual field (LVF) distractor condition. The ITC in the RVF condition in the first session and in the LVF condition in the second session did not significantly change. The concentric white circles denote *p* < 0.05 in the CBnPP. **(C,D)** Illustrate the temporal profiles of ITC in the two regions of interest (bold black circles in **(A,B)**).

To investigate whether contingent reorienting induced phase modulation, ITC was computed for each electrode. ITC reflects the consistency of oscillatory phase across trials at a particular latency. Larger ITC indicates that trials are phase-locked to each other. We used the same contrast (TC vs. NTC distractor conditions) as in the previous CBnPP test for the ITC analysis (Figure 6). In the first session, ITC increased in the left temporal and parietal regions from the onset of contralateral distractors to 300 ms after onset (Figure 6A). In the second session, the increase of ITC shifted to the right hemisphere and originated from the frontal regions (Figure 6B). The increase of ITC was only significant for distractors that appeared in the RVF in the first session and for distractors in the LVF in the second session.

### Source Estimation of Theta-Band Activity

**Figure 7:**
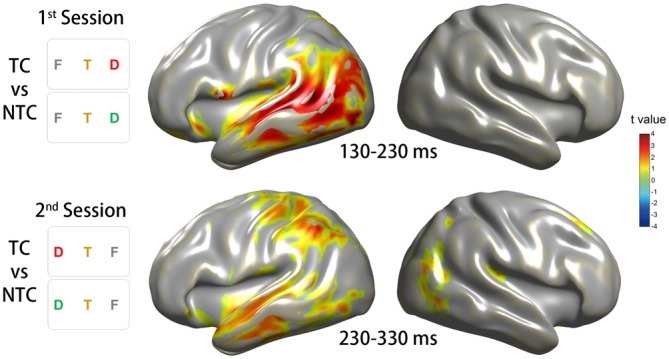
**Source reconstruction of the ITC in the theta-band activity.** The source reconstruction was based on the conditions which revealed significant ITC changes on the sensor level. The top row of the diagram illustrates the results for the CBnPP test between the right TC and NTC distractor conditions in the time window of 130–230 ms in the first session. The bottom row of the diagram illustrates the results for the CBnPP test between the left TC and NTC distractor conditions in the time window of 230–330 ms in the second session. Only the grid points with *p* < 0.05 in the CBnPP test will be illustrated in color.

To investigate the phase activity in the source level during the corresponding time window of the significant sensor-level ITC increase, we focused on the time windows of 100 ms according to the time point of strong sensor-level ITC increase obtained from the CBnPP test for the two experimental sessions. In the first session, the source phase activity of the contrast between the TC and NTC distractor conditions in the time window of 130–230 ms revealed a significant ITC increase in the left lateralized cortical area, especially in the VAN (Figure 7, top row). In the second session, the source phase activity in the time window of 230–330 ms revealed a bilateral ITC increase, including the bilateral VAN and the left DAN (Figure 7, bottom row).

## Discussion

The locus of visual attention is contingent on the interaction between top-down and stimulus-driven attentional control (Folk et al., 1992, 2002; Hickey et al., 2009; Sawaki and Luck, 2010). When attention needs to reorient from the current focus, two discrete attentional networks are recruited (Serences et al., 2005; Corbetta et al., 2008; Shulman et al., 2009; Diquattro et al., 2013). In this study, we investigated the underlying neural mechanisms of contingent reorienting from the perspectives of neural oscillations and ERPs. In line with previous findings, the task-relevant distractors induced a significant N2pc component, which indexes the locus of spatial attention. This significant N2pc component supports that the decrement of accuracy in central target identification was due to an unnecessary shift of spatial attention to the peripheral distractors. However, only oscillatory activity in the theta band revealed a dynamic temporal evolution and hemispheric variation in contingent reorienting. The ITC of theta band oscillation, which was phase-locked to the onset of contingent reorienting, demonstrated that the involvement of the two discrete attentional networks dynamically changed at different time points. The current study detailed a transition from a left attentional network to a bilateral attentional network while subjects were continuously engaged in contingent reorienting.

### Attentional Network and Contingent Reorienting

Contingent reorienting has been considered as the interaction between top-down control and stimulus-driven control that are associated with the DAN and VAN, respectively. The communication and interaction between/within the DANs and VANs is vital for online and dynamic attentional reorienting. Our results demonstrate that frequency-specific modulation in the theta band is responsible for such between-regions communication in contingent reorienting. Theta oscillation is recently regarded as a crucial index for spatial attention orienting (Daitch et al., 2013). However, the functional role of theta oscillation remains inconclusive. Several studies have indicated that the theta phase modulates the gamma power, which is related to active sensory processing. For instance, the cortical alpha/theta oscillations are predictive of the perception threshold for attended stimuli (Mathewson et al., 2009) and phosphenes (Dugué et al., 2011). Lisman and Jensen (2013) have recently proposed an oscillatory framework for this low frequency modulation. They suggested that the interaction between low and high frequencies could serve as a coding system for representing multiple items in an ordered way. The gamma frequency oscillations can be nested within slow frequency oscillations, such as alpha and theta, to represent multiple components of the different visual stimuli.

In the current experiment, the theta modulation following task-relevant distractor may index the corresponding sensory processing of the unexpected but task-relevant stimuli. The theta-band oscillations became phase-locked to the appearance of the task-relevant distractors, and this task-specific theta oscillation occurred across both the VAN and DAN. This result is consistent with the findings of a recent recording study using ECoG (Daitch et al., 2013), which demonstrated that the DANs and VANs revealed a transient phase resetting in the theta oscillation following an invalid target in the Posner attention cuing task while the subjects needed to reorient attention to an unattended location.

Whereas the prevalent hypothesis considers the VAN as right lateralized (Corbetta and Shulman, 2002; Corbetta et al., 2008), the current study indicates the possible involvement of the left VAN in contingent reorienting. Previous imaging and ECoG studies have mainly reported activity of the VAN in the right hemisphere (Shulman et al., 2010; Daitch et al., 2013; Diquattro et al., 2013). In addition, a previous study that applied offline transcranial magnetic stimulation over the bilateral VAN also supports that the right VAN but not the left VAN is crucial for attentional reorienting (Chang et al., 2013). In the present study, our observation of theta coherence in the left VAN raises the question of its functional role in attentional control. In fact, some studies reported bilateral activation in the VAN while subjects reoriented attention to the peripheral visual fields (Serences et al., 2005; Asplund et al., 2010). Such inconsistency in findings may lie in whether subjects needed to actively orient their attention to the peripheral visual fields and where they maintained their attention. When subjects needed to actively shift attention between the LVFs and RVFs without maintaining their attention at the center, activation of the left VAN was less reported (Yantis et al., 2002; Shulman et al., 2009, 2010; Diquattro et al., 2013). Furthermore, although previous evidence from off-line magnetic stimulation supports the right hemisphere dominance hypothesis (Chang et al., 2013), off-line stimulation techniques may lack sufficient temporal resolution to reveal the dynamic activity of the left VAN. Further studies combining both EEG and brain stimulation is needed to investigate the causal relationship between theta oscillation and attentional reorienting. The shift of theta coherence from the left VAN to the bilateral attentional network demonstrates that the attentional network underlying contingent capture could dynamically change with time. This shift might be highly related to practice or the deployment of top-down control. The visual attentional network exhibits right hemisphere dominance in numerous studies (Yantis et al., 2002; Shulman et al., 2009, 2010; Diquattro et al., 2013; Fellrath et al., 2014). The shift of activation to the right after practice might indicate a takeover of control by these dominant regions in the right hemisphere. A recent EEG study using a similar contingent capture paradigm indicated that, after considerable practice, the contingent capture effect was associated with the right posterior parietal cortex (Fellrath et al., 2014). It might be possible that the left VAN is activated when encountering unfamiliar task-relevant distractors.

### The Relationship Between Theta Oscillation and N2pc

In addition to the frequency domain analysis, we also applied time domain analysis to investigate the relationship between the theta IMF and electrophysiological activity in the coordination of top-down and stimulus-driven attentional networks. Although previous studies showed a relationship between theta oscillation and N2pc by showing similar onset latency (Dowdall et al., 2012; Painter et al., 2014), we found that the theta oscillation and N2pc did not totally act in the same way in terms of amplitude variation during contingent capture. In the ERP analysis of the contralateral-minus-ipsilateral difference, a positive wave appeared both immediately before and after the N2pc. Such pattern in the ERP remained the same across visual fields and experimental sessions. In contrast, although the theta IMF showed a similar pattern to the ERP in terms of waveform and time window, the amplitude of the theta IMF was modulated simultaneously by visual fields and experimental sessions.

The causes of this difference might be due to differences in the signal-to-noise ratio and in the method of analysis. While the ERP analysis integrated all frequency bands, the IMFs decomposed by EEMD were able to isolate the theta oscillation from other frequency bands and remove possible noises other than theta oscillation. This improved signal-to-noise ratio may potentially explain why the interaction was only observed in the theta IMF rather than N2pc. In terms of method of analysis, N2pc is a contralateral-minus-ipsilateral difference whereas the theta IMF is the original data obtained from each electrode. Given the difference in how the two components were obtained, it is hard to directly compare these ERP waveforms with the theta IMF. From the correlation results of N2pc and the theta IMF, the non-significant correlation might imply that the functional roles of N2pc and theta IMF are different and they are not directly associated with each other. The Np2c is an index for the locus of spatial attention whereas the theta IMF might be associated with the top-down modulation in different attentional network in the current study.

The positive contralateral-minus-ipsilateral difference waves observed immediately before and after N2pc may play a role in attentional reorienting. Because the positive waves were calculated in the same way as N2pc, they should also reflect the processing of the lateral stimuli. Considering that the peripheral stimuli in our RSVP were always distractors which occurred prior to the central target, the contralateral-minus-ipsilateral difference likely indicates the processing of the peripheral distractors. Thus, the early-onset positive-going component could reflect the early sensory processing of distractors (Mishra et al., 2012; Painter et al., 2014) or inhibition of distractors (Hickey et al., 2009; Sawaki and Luck, 2010). The subsequent N2pc signifies the locus of spatial attention, which explains why only TC distractors but not NTC distractors successfully induced N2pc in the current and previous results (Sawaki and Luck, 2010; Kiss et al., 2012). The positive wave that occurred after the N2pc could reflect disengagement from the peripheral visual fields to the central target (Toffanin et al., 2011).

## Conclusion

In summary, the present results obtained from analysis of brain waves in both time and frequency domains provide insight into the neural mechanisms of contingent reorienting. While our ERP results confirm that contingent reorienting was related to N2pc, the theta oscillation reveals that such contingent reorienting was modulated by visual fields and experimental sessions. Most importantly, the instantaneous scalp distribution of theta oscillation indicates that the task-specific and frequency-specific modulation was related to the phase-locked activity within different attentional networks. Our results demonstrate that the involvement of the attentional networks was different in the two sessions. To further elucidate the mechanisms of theta oscillation in spatial attention, brain entrainment tools, such as transcranial alternating current stimulation (Clayton et al., 2015) and frequency-based transcranial magnetic stimulation (Thut and Miniussi, 2009), would be critical for future studies. For example, a previous study already showed that contingent reorienting can be effectively modulated by applying theta-burst magnetic stimulation over the right VAN (Chang et al., 2013). Considering that theta-burst stimulation and theta oscillation share the same frequency, future research can test whether such neural modulation of theta-burst stimulation in spatial attention is related to the amplitude and coherence of the theta oscillation by combining EEG recording, transcranial alternating current stimulation and advanced time-frequency analysis.

## Author Contributions

C-FC and C-HJ study design; C-FC and C-LL collection and assembly of data; C-FC, C-LL, DLH, W-KL and C-HJ data analysis and interpretation. C-FC, W-KL and C-HJ manuscript writing.

## Conflict of Interest Statement

The authors declare that the research was conducted in the absence of any commercial or financial relationships that could be construed as a potential conflict of interest.

## Footnotes

^1^http://erpinfo.org/erplab/

^2^http://rcada.ncu.edu.tw/

^3^http://fil.ion.ucl.ac.uk/spm/

^4^http://www.ru.nl/fcdonders/fieldtrip
